# Blood safety concerns in the Eastern Mediterranean region

**Published:** 2011-06-01

**Authors:** Abdol Majid Cheraghali

**Affiliations:** 1 Iran Blood Transfusion Organization Research Center, Tehran, IR Iran; 2 Department of Pharmacology, Baqiyatallah University of Medical Sciences, Tehran, IR Iran

**Keywords:** Blood transfusion, Blood donors, Mediterranean region

## Abstract

Blood transfusion is a life-saving component of health care systems. Nevertheless, it can also be a quick and easy method of exposing patients to risks, particularly the transmission of infectious agents to recipients. Despite substantial improvements in the safety of transfusion services worldwide, the presence of paid and replacement blood donors are still of cause concern for ensuring sustainable safe blood donations. Although the Eastern Mediterranean region consists of a heterogeneous group of countries that vary in their levels of development, they all share common concerns regarding blood safety. In the region, concerns regarding the spread of Hepatitis B and C through blood transfusion continue to exist. Therefore, there is an urgent need for further improvements in both organization and safety measures of blood transfusion activities in the region. Although establishing a centralized blood transfusion system might not be achievable in the short term in some of the countries in the region, the implementation of centralized test kit procurement, data collection, and donation testing could be considered feasible approaches.

## 1. Background

Presently, blood transfusion is an essential component of modern health care that saves millions of lives each year, and it will continue to be so for many years to come. Every second, someone in the world needs blood for surgery, trauma, severe anemia, or complications from pregnancy. According to a World Health Organization (WHO) estimate, each year over 100 million people sustain injuries and more than five million die from violence and injury. Annually, worldwide, more than 536,000 women die during pregnancy or childbirth; 99% of these cases are in developing countries, and most mortalities are due to bleeding [1]. Therefore, the availability of timely and safe blood transfusions in health care facilities providing emergency obstetric care can entail the difference between life and death. Although in developed countries, transfusion of blood and blood components is mostly used to support advanced medical and surgical procedures, in resource-limited countries, the majority of blood and its components are used for the management of obstetric emergencies and children suffering from severe anemia. Blood transfusions can also be a quick and easy way of exposing risks, such as transmission of infectious agents like HIV, HBV, HCV, and malaria to the recipients. Despite considerable improvements in blood transfusion safety measures in developing countries, blood transfusion still accounts for a substantial portion of HIV transmissions in developing countries. Known and emerging threats to blood safety and the balance between the impact of measures to contain these threats, and the adequacy and accessibility of blood and blood components require national coordination and planning. Every nation should meet its requirements for blood and blood products and ensure that blood supplies are practically free from life-threatening transfusion-transmissible infections (TTIs).

Unfortunately, millions of patients living in developing countries do not have timely access to safe blood. Although more than 80% of the world's population lives in the developing world, less than 45% of over 100 million units of blood donated globally are collected in these countries. The average number of blood donations per 1,000 persons is 10 times higher in high-income countries than low-income countries. An inadequate stock of blood usually forces health providers in low-income countries to turn to unsafe replacements or paid donors, thus increasing the risk of using with questionable safety. Lack of qualified organizational practices such as fragmentation and low efficiency of blood services' operations, as well as lack of tangible political commitment and support, are the main challenges in developing countries regarding blood safety. These usually lead to difficulties in ensuring the sustainability of blood services. In order to improve blood safety and the availability of blood components, the WHO has proposed a five-tiered strategy for the establishment of a national blood transfusion policy and service. The main components of this strategy are as follows [2]:

-The establishment of well-organized, nationally coordinated blood transfusion;

-The collection of blood from voluntary non-remunerated blood donors;

-Quality-assured testing for TTIs and compatibility testing;

-The safe and appropriate use of blood;

-Quality systems covering the entire transfusion process.

Sadly, despite a markedly inadequate blood supply in many countries, especially in the developing world, even when simpler and less expensive interventions with equal benefit are available, the inappropriate use of blood transfusions will push the availability of the blood components to its limit. Citizens in developing countries might not even have universal access to blood transfusions. In these countries, the development of blood transfusion services has been largely restricted to major cities, and people living in rural and remote areas mostly lack access to safe blood.

## 2. Global status of transfusion safety

The following are the findings of a survey published by the WHO in 2010 [3] on the basis of data collected from 173 countries that received 93 million blood donations:

41 countries were unable to screen all blood donations for one or more of the following TTIs, including HIV, HBV, HCV, and syphilis. Only 47% of blood donations in developing countries are screened following basic quality assurance procedures. Of the 93 million donations, 50% were collected in developed countries, home to 16% of the world's population.

It is estimated that donations from 1% of the population is generally the minimum needed to meet a nation's most basic requirements for blood. The average donation rate is 3% in developed countries, 0.75% in transitional countries, and an average of 0.37% in developing countries.

Although 56 countries reported more than a 10% increase in voluntary unpaid blood donations, 15 countries reported more than a 10% decrease in this figure compared to the previous year's figure.

The availability of blood with compromised safety might expose recipients to avoidable life-threatening risks. The transmission of viral and other infections like syphilis and malaria are among the most important risks when receiving tainted blood. However, there are also concerns regarding the transmission of emerging diseases through blood transfusion. An unsafe blood supply represents the major contributor of the transmission of HCV, especially in many developing countries [4]. It is also estimated that blood transfusion may account for up to 15% of HIV transmissions in developing countries[5]. To improve blood safety, the aim should be to reduce the number of transfusions to the minimum whenever feasible and to use blood substitutes wherever available.

## 3. Status of blood safety in the region

The Eastern Mediterranean region consists of a heterogeneous group of countries with various levels of development. However, they all share common concerns regarding blood safety. In the 1990s, blood transfusions was reported to be one of the main causes of HIV contamination in the Middle East[6]. A cumulative total of 3,745 AIDS cases were reported from the countries of the region through the end of 1995. Information about the mode of transmission was available in 3,461 cases (92.4%). Of that percentage, 368 (10.6%) cases were due to the receipt of blood or blood products, the largest numbers of which were reported in Iran, Morocco, Saudi Arabia, Egypt, and Iraq. However, it was later proven that the majority of the HIV cases in Iranian patients who received blood products were attributed to imported concentrated coagulation factors[7]. Nonetheless, owing to improvements in transfusion safety in the region, blood transfusion is no longer the main route of HIV transmission.

Despite recent improvements in the safety of transfusion services in the region, concerns regarding the role of blood transfusion in the spread of hepatitis B and C in the region continue to exist[8]. Therefore, WHO has urged regional member states to ensure transfusion safety by promoting safe blood donation, strengthening national regulatory activities related to quality assurance, and assuring the safety of blood products[8]. Although there is established data confirming that the prevalence of HIV, hepatitis viruses, and other TTIs are lowest among unpaid blood donors who give blood voluntarily, several countries in the region still rely heavily on remunerated donors and/or replacement donors. Despite the presence of a centralized blood transfusion service in some countries of the region, such as Iran, several countries show a high degree of fragmented blood transfusion services. Inappropriate clinical use of blood and blood products is also a major regional problem[1].

A few number of English published data is available that describes blood safety statuses in the region. This type of data is even scarcer with regard to Arab countries of the Persian Gulf coasts. According to a published survey from 17 Arab countries, 14 had thus far formulated a national policy but only nine had national regulation. In 2004, a total of 2,400,000 units of blood were collected in these 17 countries. The proportion of blood collected on the basis of voluntary blood donation in these countries varies drastically and is reported to be between 3% and 100%. Donation rates vary from 0.2% (Syria) to 2.7% (Kuwait)[9]. However, all this donated blood is tested against hepatitis B and C and HIV in these countries. Multi-transfused patients, including thalassemia patients, are among the most vulnerable to TTIs. Therefore, the prevalence of infectious agents in this group of patients could be considered an indirect index of blood safety. The prevalence of HCV infection in thalassemia patients in the Eastern Mediterranean region has been recently reviewed[10].

Although up to 80% of adult thalassemia patients are infected with HCV in the world, there is substantial variation among the countries in the region. For example, in Iran, HCV infection rates range from 2% to 32% and in Saudi Arabia, from 33% to 93%. In Kuwait and Jordan, all the HCV-infected thalassemia cases were transfused before the onset of blood donor screening. Among the major countries in the region, Iran showed the least seroprevalence of HCV infection among thalassemia patients. This underscores the more advanced blood safety in this country. However, in this regard, there is no data from most of the countries in the region[10]. In Egypt, sexual contact is the main cause (71%) of HIV transmission. However, cumulatively and until 2009, blood and blood products were identified as the cause of infection in 5% of the cases[11]. To prevent HIV transmission through blood and blood products, a blood safety policy applies to all blood banks in Egypt, which includes the screening of donated blood for HBV, HCV, HIV, and syphilis. From 2008 and 2009, 52 HIV-positive blood bags were detected and discarded. In 2009, a total number of 1,280,000 blood units were screened and 44 HIV-positive blood bags were detected and discarded[11]. An analysis of data from 211,772 blood samples collected from 2000 to 2008 in Egypt[12] has shown that the overall HBsAg and anti-HCV prevalence were 1.65% and 9.02%, respectively. Anti-HCV and HBsAg prevalence has dropped from 11.06% (in 2000) to 6.3% (in 2008) and from 1.24% (in 2000) to 1.17% (in 2008), respectively. The prevalence of anti-HCV and HBsAg was significantly higher among males donors (10% and 1.74%, respectively) compared with females (3% and 1.07%, respectively). It was also reported that there was a significantly higher occurrence of both anti-HCV and HBsAg prevalence among donors from rural area (11.3% and 2.27%, respectively) compared with urban donors[12]. Kuwait's central blood bank is the only public supplier of blood and blood components in Kuwait that performs blood and blood component collection, processing, testing, distribution, and transfusion services for all governmental and private hospitals in the country. As a reflection of the country's multinational residences, over the past decades only 40-43% of blood donors in Kuwait were Kuwaiti nationals. On the basis of a study conducted in 2002 among 26,874 blood donors to Kuwait's central blood bank, 51.2% were Kuwaiti nationals and 48.8% were non-Kuwaiti Arabs. However, only 15.3% of donations were volunteers, while 84.7% were replacement donors[13].

The prevalence of anti-HCV among Kuwaiti national and non-Kuwaiti Arab first-time donors was 0.8 and 5.4%, respectively, whereas the prevalence of HBsAg was 1.1 and 3.5%, respectively. Among the first-time donors, 13.7 percent were positive for the presence of anti-HBs[13]. These figures show that the heterogeneity of the population living in Kuwait, and the primary reliance on replacement blood donors, might have a significant impact on the prevalence of hepatitis infection among blood donors. In Iran, blood transfusion is an integral part of the national health system, and blood donation is voluntary and non-remunerated. In 1974 and following the establishment of the Iran Blood Transfusion Organization (IBTO), all blood transfusion activities-from donor recruitment to production of blood components and delivery of blood and blood products-were centralized. In order to meet the country's demand in 2010, the IBTO collected more than 1.8 million units of blood for the country's population of 74 million. Continued donor recruitment efforts in Iran have resulted in a significant increase in blood donation rates during the past decade, excluding "family replacement" donations, and achieved a 100% voluntary non-remunerated blood donation rate in 2007 [14][15][16].

The prevalence of HBsAg has been evaluated in blood donations in Iran over the period from 1998-2007[17]. A total of 14,599,783 donations were collected during these 10 years. The overall HBsAg prevalence rates declined from 1.79% in 1998 to 0.41% in 2007 as a result of improvements in donor recruitment and selection, implementation of automation in transfusion services, and a possibly decreasing HBV infection prevalence in the general population. A separate report has also examined the results from the viral screening of 6,499,851 donations from 2004 through 2007 for HBV, HCV, HIV, and syphilis[18][19]. The overall prevalence of these viral markers was 0.56% for HBV, 0.004% for HIV, and 0.13% for HCV. There was a significant decrease in HBsAg prevalence from 0.73% in 2004 to 0.41% in 2007. Despite an increasing HIV prevalence in the general population of Iran, the prevalence of HIV decreased from 0.005% in 2004 to 0.004% in 2007. HCV prevalence showed a slight decline in blood donations from 0.14% in 2005 to 0.12% in 2007.

The situation in Pakistan with regard to blood transfusion has remained far from satisfactory over the years[20][21][22][23]. There is extreme fragmentation and rampant commercialism with questionable transfusion practice quality in the majority of blood establishments throughout the country. The blood transfusion services in Pakistan are mostly hospital-based, and majority of blood donors are usually first-time replacement donors or paid and directed donors. About 75% of blood donations in Pakistan come from replacement donors, and around 15% of the blood is still donated by professional donors. Only 10% of blood donors in Pakistan make voluntary unpaid donations. It seems the cultural and socioeconomic factors are associated with a reluctance to donate blood at all, especially without reward. The annual estimated requirement of blood is approximately 1.5 million units, with 40% of the demand being met by the public sector. Although there is about a 40% shortage of blood and blood components in this country, about 85% of blood used as whole blood products [20].

In Pakistan, crude low-sensitivity kits, available at very low prices, are being used for screening. Commercial blood donors with infections like HIV, HCV, and HBV are not stringently screened before a blood donation. Published reports indicate discouraging trends in appropriate screenings of potential donors for viral infections, unsafe sexual behavior, and drug abuse[22]. Data on the serologic testing of blood donors for HBV, HCV, HIV, and syphilis in a 10-year period (1996-2005) in Lahore has been reviewed. The frequency of serologic markers ranged from 1.46-2.99% for HBV, with a downward trend over time: 3.01-4.99% for HCV, 0-0.06% for HIV, and 0.19-0.57% for syphilis[23]. However, volunteer donors (6.98% of all the donors) had the lowest seroprevalence for the diseases.

The statuses of blood transfusion services in Afghanistan have also been reviewed recently [24]. Owing to the lack of appropriate infrastructure and resources, the national blood supply system is damaged and heavily disrupted. Afghanistan, with an estimated population of 30 million, needs at least 300,000 units of blood. Presently, more than 95% of blood donation in Afghanistan comes from family replacements, which are often paid by the patient's family, and less than 5% are from voluntary donors.

## 4. Conclusion

There is a consensus that a centralized blood transfusion service has the ability to improve the availability of safe blood and blood components. Although this might be a far-reaching goal in some countries of the region, the implementation of centralized test kits, procurement and donation testing could be considered feasible approaches. Usually, a fragmented blood supply system with varying technical standards and no central supervision might disrupt safety measures. Published data regarding blood safety in the region is scarce and primarily consists of either one or a limited number of blood centers with a small number of blood samples. Therefore, it would be difficult to extrapolate results from such studies to the national level. The establishment of a data collection center at a national level, even in countries without centralized blood transfusion services, would be essential for analyzing data regarding the current blood safety status and future planning in regional countries.

Collecting blood only from voluntary donors might not be an attainable goal, at least in the short-term, for many developing countries[25], including some countries in the region. Therefore, it seems that national policymakers in these countries, while establishing a long-term plan to attain 100% voluntary blood donations, should implement a plan to wisely use replacement donors for providing safe blood to patients in need of blood and blood products. Since 2004, Iran, in a unique experience in the region, has established a long-term self-sufficiency plan for plasma-derived medicines using plasma recovered from donated blood to the national blood centers. The implemented plasma contract fractionation activity has remarkably improved quality assurance systems in Iran's national transfusion service, which ultimately has improved both national blood safety and the affordability of plasma-derived medicines[7][26]. There is strong evidence that this approach could be emulated by other countries in the region.

In conclusion, the people of the Eastern Mediterranean region, regardless of their wealth or social status, need safe blood transfusions in situations where transfusion is a life-saving procedure. Therefore, equity in the availability of blood for any citizen could be considered a development index in every country's health sector.
